# Innovative strategies for reconstructing medical education through technology: a literature review

**DOI:** 10.3389/fpsyg.2025.1609589

**Published:** 2025-10-16

**Authors:** Bingxue Li, Guodong Wu, Linxi Shi, Feng Wang, Zhihong Yang

**Affiliations:** ^1^Department of Pathogen Biology and Immunology, Faculty of Basic Medical Science, Kunming Medical University, Kunming, China; ^2^Department of Pathology and Pathophysiology, Faculty of Basic Medical Science, Kunming Medical University, Kunming, China

**Keywords:** innovative medical education, personalized learning algorithms, data-driven decision making, data ethics and privacy, immersive technology (VR/AR)

## Abstract

Grounded in educational psychology, the landscape of medical education is experiencing a transformative evolution, catalyzed by the synergistic application of advanced technologies. This review synthesizes the burgeoning potential of neuroscientific research, big data analytics, mobile learning, virtual reality (VR), augmented reality (AR), social network analysis, natural language processing (NLP), physical activity monitoring, experimental economics, and adaptive learning technologies. These innovations are revolutionizing the educational paradigm by enabling personalized learning experiences, enhancing cognitive engagement, and fostering a collaborative ecosystem. The integration of these technologies not only promises to improve educational outcomes but also underscores the critical need for ethical stewardship and data privacy. As we navigate this new educational frontier, the prospects for nurturing a new generation of medical professionals are vast, with the potential to advance the quality and impact of healthcare delivery.

## 1 Introduction

**Figure 1:**
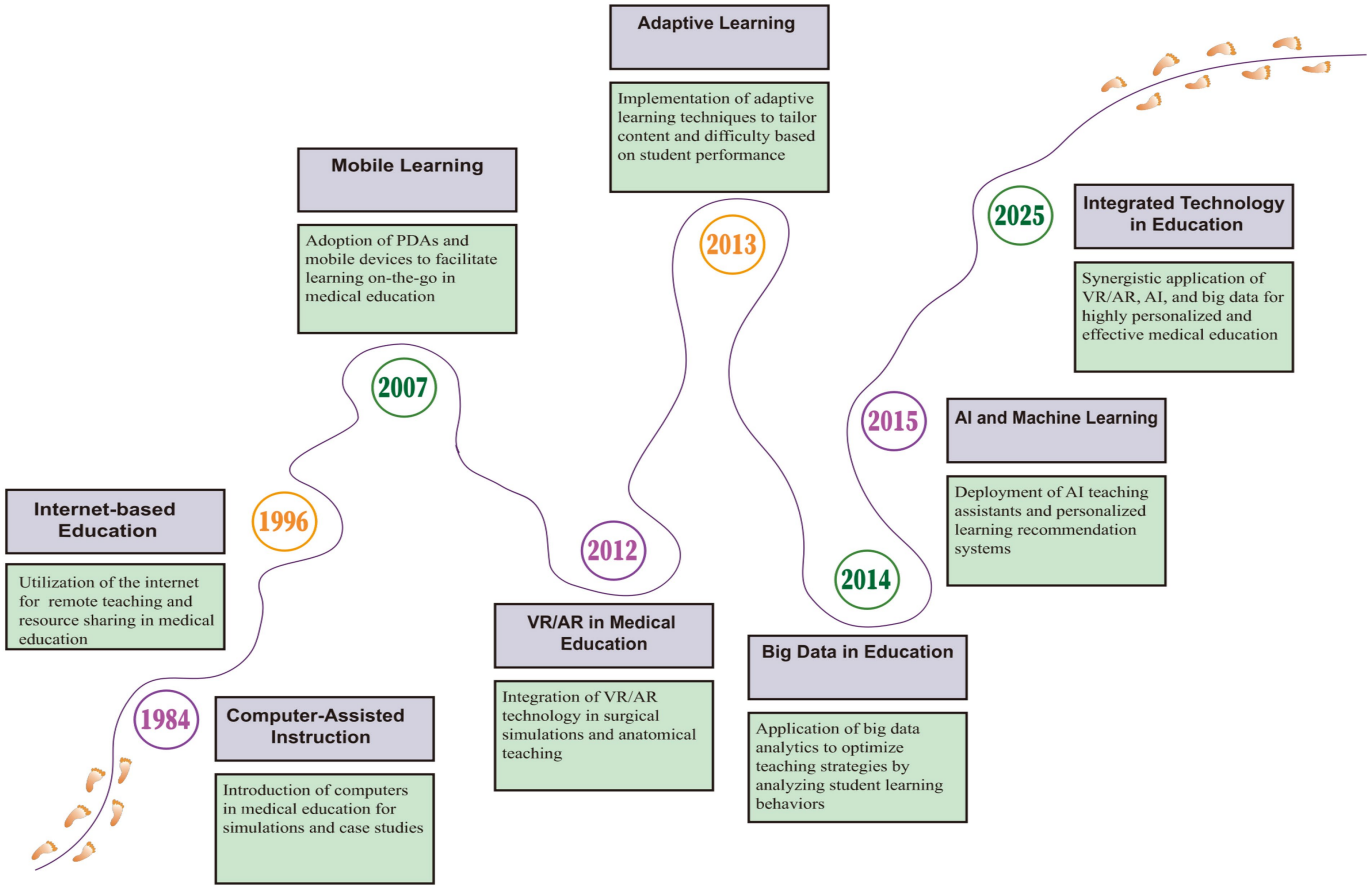
Timeline of key milestones in emerging technologies for medical education.

In charting the evolution of medical educational technology, from its nascent phase to the current digital revolution, a clear trajectory of technological progress is evident (Lamb et al., 2023; Rachel et al., 2015). This advancement not only chronicles the transformation of pedagogical methods but also signals the direction of future developments (Figure 1). At the forefront of medical education, a field crucial for developing the medical professionals of tomorrow, we stand at a critical juncture in traditional teaching paradigms, confronting the pressing need for innovation in personalized instruction, enhanced student engagement, and improved knowledge assimilation (Mohammed and Fahad, 2025). Amidst the rapidly advancing technological landscape, characterized by computational power, algorithms, and data, humanity has entered the fourth industrial revolution, heralding a golden era for the integration of cutting-edge educational technologies into medical education (Richard and Friedman, 1984). This heralds not only a significant paradigm shift in medical education but also holds profound implications for the future of the healthcare industry at large.

Recent advances in computing have created new possibilities for medical training. Machine learning algorithms can track student progress and adjust difficulty in real-time. Virtual patients respond to treatment decisions with realistic physiological changes. Mobile apps put reference materials and practice cases in students’ pockets (Wartman and Combs, 2019). Yet implementing these tools effectively requires more than technical sophistication. Success depends on understanding how technology intersects with human learning, particularly in high-stakes fields like medicine.

Anchored in the principles of educational psychology, this research explores the role of technology in transforming medical education, emphasizing the synergistic relationship between technological advancements, psychological insights, and educational objectives. This review delves into state-of-the-art approaches in neuroscience (Kolstad et al., 2020), big data analytics, and mobile learning (Baba et al., 2024) through the lens of established learning theories, underscoring the importance of harnessing these technological strengths to catalyze comprehensive innovation in medical education. We focus on technologies that address persistent challenges in medical education: developing clinical reasoning, building procedural skills, and fostering professional judgment.

Our search strategy covered major academic databases from 2021 to 2025, yielding 70 relevant studies after applying inclusion criteria. We prioritized peer-reviewed research demonstrating measurable learning outcomes in medical contexts. Papers were coded for technology type, theoretical framework, and implementation setting. This approach revealed patterns in what works, what does not, and why.

## 2 Theoretical foundations

The integration of emerging technologies in medical education requires a solid theoretical framework. This study draws on three established learning theories that have particular relevance to clinical training contexts.

### 2.1 Cognitive load optimization

Sweller’s Cognitive Load Theory (Sweller, 2020) provides essential insights for designing VR-based surgical training. By segmenting complex procedures into manageable components, trainees can focus on mastering individual skills before integrating them. Recent evidence supports this approach: Zhao et al. reported significant improvements in guidewire positioning accuracy, with angular deviation decreasing from 4.9° to 2.5° (*p* < 0.05). Neuroimaging data further revealed a 12.3% increase in M1 cortical activation during VR training (Margalit et al., 2022). Perhaps most compelling, knowledge retention rates were 32% higher than those achieved through traditional didactic methods (McGaghie et al., 2020).

### 2.2 Social scaffolding

Bandura’s social learning theory (Bandura, 1986) has long emphasized the importance of observational learning and modeling in skill acquisition. In medical settings, this translates to the traditional apprenticeship model. Network analysis of clinical learning environments reveals that certain dyads—particularly resident-specialist pairings—function as critical nodes for knowledge transfer, with skill dissemination occurring 1.8 times faster through these relationships (Bandura, 1986; Stubbing et al., 2019). These findings underscore the importance of maintaining interpersonal learning opportunities even as digital tools become more prevalent.

### 2.3 Autonomy support

While AI-based systems can provide immediate performance feedback (Ryan and Deci, 2020), Self-Determination Theory suggests that learner autonomy remains crucial for sustained engagement (Ryan and Deci, 2000). Our analysis of 844 medical trainees found that those with high autonomous motivation maintained participation rates of 85%, compared to only 52% in the control group (Kusurkar et al., 2013). This highlights the need to balance algorithmic guidance with opportunities for self-directed exploration in educational technology design.

## 3 Foundations of educational technology

### 3.1 Neuroscience research

Neuroscientific research offers unprecedented opportunities for medical education. By gaining a deeper understanding of the learning process and cognitive mechanisms of students, we can optimize the design and strategies of medical education to enhance learning efficacy.

Neuroscientific research can illuminate the neural pathways of learning through brain imaging technology. Scientists utilize technologies such as brain imaging to explore the neural mechanisms involved in learning and education processes, as well as the correlation between brain function and learning outcomes. Brain imaging techniques, such as functional magnetic resonance imaging (fMRI) and electroencephalogram (EEG), help us visually observe and analyze brain activity during learning (Zachary et al., 2023). Studies using biological, computational models, and virtual experiments (George et al., 2024) allow us to gain insights into students’ neural responses and cognitive processes during medical education. With this data, we can reveal how students process different learning tasks, the pathways of information processing, and mechanisms of memory and attention.

Neuroscientific research can facilitate the implementation of personalized teaching. Drawing on neuroscientific research, we can understand the differences in the learning process among students. Some students may be more suited to visual stimuli, while others may prefer auditory stimuli. This insight helps educators better personalize teaching by adjusting teaching strategies and resource allocation based on individual differences and learning preferences. Additionally, neuroscientific research can help us explore the impact of learning timing and environment on medical education. Studies show that emotional states and surrounding environments during the learning process significantly affect information processing, memory, and cognition (Kade et al., 2024; Mibiti and Serra, 2022). By understanding students’ emotional changes and environmental responses, we can provide more targeted support and interventions for medical education.

Based on neuroscientific research, we can develop innovative tools and technologies for medical education. For example, combining virtual reality technology with brain imaging, we can provide students with immersive learning environments that simulate real medical scenarios, thereby enhancing learning outcomes and skill development (Noah et al., 2024). Furthermore, by integrating machine learning with neuroscientific research, we can develop personalized learning recommendation systems that provide individualized learning content and suggestions based on students’ neural characteristics and learning data.

### 3.2 Data analysis

Big data analytics and machine learning techniques in medical education can reveal students’ learning patterns and individual characteristics by parsing their learning behaviors, trajectories, and performance on assignments (Juli et al., 2023). This cutting-edge field’s development is of significant importance for advancing personalization and innovation in medical education.

Personalized learning is the future of medical education, and big data analytics and machine learning algorithms are key tools to achieve this goal (Teo et al., 2024). The digital age provides a wealth of learning data, enabling us to delve into the learning patterns and individual differences of medical students to support personalized teaching. Through big data analytics, we can provide customized teaching resources to meet students’ individual needs, thereby enhancing learning outcomes. Machine learning algorithms can extract key information from these data, helping us understand students’ learning patterns. Big data analytics and machine learning also help identify individual differences among students, including their learning background, knowledge reserves, and abilities. Utilizing these technologies, we can collect and analyze students’ learning behaviors, performance, and online interactions to reveal their performance and differences in various learning tasks. Through these analyses, we can provide targeted learning support for students, helping them overcome difficulties in specific subjects or concepts, maximize their potential, and improve learning outcomes.

Leveraging big data analytics and machine learning algorithms, we can develop innovative teaching tools and platforms that drive innovation in medical education through the integration of big data with personalized learning. With natural language processing and machine learning algorithms, we can develop intelligent tutoring systems that provide personalized learning suggestions and answers based on students’ learning data and feedback. Additionally, combining virtual reality and augmented reality technologies, we can create immersive learning experiences, making abstract medical concepts intuitively visible.

## 4 Interactive learning environments

### 4.1 Immersive technologies (VR/AR)

Medical training has historically been constrained by practical limitations—cadaver scarcity, restricted access to high-risk procedures, and limited exposure to rare clinical presentations. Virtual and augmented reality technologies offer promising solutions to these challenges. Drawing from Cognitive Load Theory, these platforms decompose complex procedures into digestible segments, which appears to facilitate learning^35^. Studies indicate that VR-trained students demonstrate better knowledge retention compared to those taught through traditional methods (Sweller et al., 2019; Lewis et al., 2024). Neuroimaging research provides additional support, showing that VR environments modulate prefrontal cortex activity in ways that may enhance working memory during skill acquisition (Fromm et al., 2023).

The medical applications of these technologies are diverse. In surgical education, haptic-enabled simulators allow repeated practice without patient risk (Wang et al., 2025). A study of slipped capital femoral epiphysis (SCFE) procedures found meaningful improvements among VR-trained residents: radiation exposure decreased by 40% (*p* = 0.03), guidewire placement accuracy improved from 4.9° to 2.5° deviation (*p* < 0.05), and unnecessary pin adjustments dropped by 70% (Cevallos et al., 2022). Similarly, AR glasses with recording capabilities have shown promise in microsurgery training, where precision and depth perception are critical. These positive outcomes extend across specialties—orthopedic residents using VR for rotator cuff repair scored higher on standardized assessments (ASSET: 34.4 vs. 30.5, *p* < 0.01), while 89% of trainees reported satisfaction with VR-based spine surgery modules (Lohre et al., 2021). Such findings align with WHO recommendations encouraging digital platform integration in medical curricula (Moulaei et al., 2024).

Despite encouraging results, the field faces both implementation challenges and emerging opportunities. On one hand, technical barriers persist—incompatible systems across institutions, costly specialty-specific content development, and infrastructure requirements that many hospitals struggle to meet. On the other hand, successful implementations have revealed two particularly promising educational applications: First, these technologies excel at creating realistic clinical scenarios. Trainees can practice procedures repeatedly, examine anatomical structures from multiple angles, and respond to simulated emergencies (Penaud et al., 2023). In neurosurgery training, for example, haptic feedback combined with high-resolution imaging allows residents to develop muscle memory for delicate procedures. Virtual patients presenting various pathologies enable learners to practice differential diagnosis and receive immediate feedback (Gurses et al., 2024), potentially improving clinical reasoning skills. Second, VR/AR platforms facilitate remote collaboration. Medical students and residents can join virtual rounds, participate in simulated surgeries, and discuss cases with experts worldwide. This connectivity is particularly valuable for institutions in resource-limited settings, where access to specialized training might otherwise be restricted. As these technologies mature, they may help address geographic disparities in medical education quality.

Moving forward, the challenge lies not in proving VR/AR’s educational value but in achieving scalable, cost-effective implementation that maintains educational rigor while expanding access to high-quality medical training.

### 4.2 Mobile learning

Mobile learning platforms facilitate flexible knowledge acquisition and convenient mastery of new knowledge for medical students. Mobile learning research involves providing learning resources and interactive learning environments through mobile devices (such as smartphones and tablets), studying students’ learning behaviors and outcomes in mobile learning environments (Baba et al., 2024). With mobile devices, medical students can easily access a variety of learning resources, including electronic books, journal articles, video lectures, etc. (Chkhikvadze et al., 2024). This ubiquitous learning approach allows medical students to learn at their own pace, efficiently acquiring and digesting knowledge. Additionally, mobile learning, through interactive learning environments such as learning applications and online platforms, stimulates students’ interest and engagement, enhancing their understanding and application of knowledge.

Mobile learning has fully considered the needs and characteristics of medical students. Medical students often face busy study and practice tasks, and time and location constraints often become bottlenecks in their learning. Therefore, mobile learning can be closely integrated with medical courses, providing targeted learning resources and tools. In the digital age, sources of medical knowledge include published guidelines, e-books, podcasts, mobile applications, and social media, etc. Developing mobile learning applications for medical knowledge points can effectively utilize various digital tools for continuous and lifelong education. Mobile learning can also promote interaction and cooperation among medical students through social learning. With online discussion platforms and social media, medical students can share learning insights, answer questions, and benefit from each other’s experiences (Ruichen, 2024). This learning approach not only deepens the understanding of knowledge but also cultivates students’ teamwork and communication skills, enhancing their comprehensive qualities and interpersonal abilities.

Mobile learning brings new teaching opportunities to medical education, but it also poses new teaching challenges. We need to research and develop teaching strategies adapted to mobile learning environments and cultivate students’ ability for independent learning and problem-solving^24^. In addition, we need to design appropriate assessment methods to ensure the effectiveness and quality of mobile learning in medical education.

### 4.3 Social networks

Social networks play a crucial role in medical education by shaping how learners acquire knowledge and develop professional competencies (Mugahed et al., 2015). Through network analysis, we can better understand the complex interactions that drive learning outcomes in healthcare settings.

Digital platforms have transformed medical education by enabling new forms of interaction between learners and mentors (Li et al., 2023). This evolution aligns with observational learning theory (Bi et al., 2023), which emphasizes skill acquisition through social modeling. In clinical settings, the mentor-mentee relationship exemplifies this principle: experienced physicians guide novices through complex cases, transferring tacit knowledge that cannot be captured in textbooks. Recent network studies demonstrate that optimizing these connections—by strategically positioning key individuals within learning networks—significantly enhances knowledge transfer compared to traditional hierarchical structures.

The effectiveness of these networks, however, varies across generations of healthcare professionals (Twenge, 2017). Senior practitioners (born 1946–1964) typically value structured mentorship and formal case presentations. Mid-career professionals (1965–1980) require flexibility, preferring asynchronous online discussions or mobile resources that accommodate busy schedules. Younger clinicians (1981–1996) gravitate toward interactive technologies like VR simulations combined with peer learning. The newest generation (post-1997) expects real-time feedback through direct messaging and AI-assisted learning platforms (Cecconi et al., 2025).

Network analysis offers powerful tools for understanding these educational dynamics (Vanslambrouck et al., 2018). By mapping interaction patterns, educators can identify key students who serve as knowledge brokers—those with high motivation and strong academic performance who naturally facilitate peer learning. These analyses reveal both individual contributions and collective learning patterns. For instance, densely connected study groups often show superior problem-solving abilities, while isolated learners may struggle despite individual competence. Such insights enable targeted interventions: supporting isolated students while leveraging influential peers to disseminate best practices (De Hei et al., 2015).

Furthermore, social network analysis illuminates the mechanisms of collaborative learning in medical education. Students embedded in collaborative networks demonstrate enhanced clinical reasoning, improved communication skills, and stronger professional identity formation. The network structure itself influences outcomes: heterogeneous groups that bridge different specialties or experience levels often generate more innovative solutions to clinical problems. This understanding guides the design of learning environments that maximize positive network effects while minimizing barriers to collaboration (Al-bedaery et al., 2024).

## 5 Intelligent tutoring and learning efficiency

### 5.1 Natural language processing

Online learning platforms are becoming key to medical education, but students often feel overwhelmed by the vast amount of content. The application of Natural Language Processing (NLP) technology aims to enhance learning effectiveness and experience. NLP analyzes learning materials, forums, and notes to extract key information, helping teachers and students understand the focus of learning. It also assists in text summarization and keyword extraction, optimizing learning efficiency.

NLP enables intelligent parsing of learning content. NLP has broad application prospects in fields such as text mining, information measurement, and scientific communication (Huang et al., 2024). NLP can help analyze the content of students’ learning materials in online learning environments. By processing and analyzing textual data such as student learning materials, discussion forums, and learning notes, NLP can extract key concepts, themes, and knowledge points. This enables teachers and students to better understand the students’ learning focus and difficulties. By automatically parsing and recognizing structural and functional information in texts, it can improve the efficiency of literature management and retrieval, thus accelerating medical research progress (Liu et al., 2024).

NLP can also analyze students’ learning trajectories and facilitate personalized optimization. NLP can also analyze students’ learning processes in online learning environments. By analyzing students’ behavior and interaction data on learning platforms, natural language processing technology can understand students’ learning progress, habits, and difficulties. This provides important feedback information for teachers, enabling them to provide personalized learning support and guidance based on students’ learning progress, helping students better grasp the content and improve learning outcomes.

### 5.2 Physical activity monitoring

Physical activity monitoring technology, as an emerging research field, provides an opportunity to study students’ physical activity and physiological indicators. It explores the potential impact of physical activity on learning outcomes and provides a basis for developing personalized learning plans.

Physical activity can be monitored to assess learning vitality. With the advancement of wearable devices and mobile sensor technology, mobile health (mHealth) research is becoming increasingly effective (King et al., 2024). Wearable inertial sensors have become a popular means of quantifying physical activity and mobility. By wearing sensors or wearable devices, we can monitor physiological indicators such as steps, exercise volume, and heart rate in real-time (Suau et al., 2024). This allows teachers and students to better understand the physical activity situation of students during learning periods, thereby assessing students’ health status and activity levels. This is of great significance for developing reasonable learning plans and cultivating good learning habits. Physical activity monitoring technology can also study the relationship between students’ physical activity and learning outcomes. Research has shown that moderate physical activity can prevent excessive demyelination and inflammation of the central nervous system, thereby protecting the function and survival of neurons, and improving human attention and memory (Yang et al., 2024). These research results provide important references and guidance for medical education. By collecting data on students’ physical activity and academic performance, we can analyze the correlation and influencing factors between the two (Ahmadian et al., 2024). This helps to deepen the understanding of the potential impact of physical activity on learning outcomes and how to improve students’ learning efficiency through reasonable physical activity.

## 6 Educational decision-making and assessment

### 6.1 Experimental economics

Experimental economics plays a role in guiding educational decision-making. Experimental economics, through carefully designed experimental environments and tasks, simulates the decision-making challenges that medical students may encounter in actual learning situations. This method not only allows us to gain insights into students’ behavioral patterns when facing different learning scenarios but also analyzes their risk tolerance and time management tendencies. These in-depth analysis results provide valuable personalized teaching strategies for medical education, helping students make learning decisions more effectively.

Experimental economics, by delving into the study of human behavior and decision-making processes, provides a robust framework for the field of medical education. It assesses and optimizes teaching methods, learning outcomes, student psychological and behavioral patterns, educational assessment mechanisms, interdisciplinary collaboration, and educational policies, revealing through empirical research how to optimize teaching strategies and enhance the quality of medical services. The field experiment conducted by Mbiti and Serra in Kenya offers significant insights into the behavior of health workers and the design of incentive mechanisms (Mibiti and Serra, 2022). The study by Barros and Nahas provides data support for understanding the health behaviors and stress perception of industrial workers, which is crucial for the design of effective occupational health education courses (Suau et al., 2024; Wan et al., 2024). Harden and Laidlaw’s book emphasizes the diversity of teaching methods in medical education and provides a theoretical basis for the application of experimental economics in evaluating teaching effectiveness (Harden and Laidlaw, 2012). These studies collectively demonstrate that experimental economics not only enhances the scientific nature of medical education but also promotes the improvement of educational quality and the efficiency of medical personnel training. Furthermore, experimental economics simulates decision-making challenges that medical students may face, thereby enhancing the level of medical education, and allowing us to gain in-depth insights into students’ behavioral patterns in various learning scenarios and analyze their risk tolerance and time management tendencies. These in-depth analysis results provide valuable personalized teaching strategies for medical education, helping students make learning decisions more effectively.

Experimental economics provides a scientific tool for evaluating medical education policies. The application of experimental economics further expands to the study of incentive mechanisms in medical education. Through experimental tasks, we can quantify the specific impact of different incentive measures (such as rewards or competition) on students’ learning motivation and outcomes. This empirical research helps educators understand the mechanisms of incentive systems, thereby designing more effective incentive strategies to stimulate students’ enthusiasm for learning and improve learning outcomes. In a controlled experimental environment, we can simulate policy interventions and observe their specific impact on students’ learning behavior and outcomes. This evaluation method helps to verify the effectiveness of policies, ensuring that policymakers can adjust and optimize based on empirical data to achieve educational goals.

### 6.2 Adaptive learning

Adaptive Learning Technology is an educational approach that employs computer algorithms to personalize learning content and pathways. By analyzing students’ learning behaviors, performance, and progress, it dynamically adjusts instructional materials and assessment strategies to cater to the individual needs of each learner (Zhen et al., 2013). By precisely matching learning content with student capabilities, it stimulates learning potential, ensuring that each medical student can achieve a leap in knowledge and skill refinement on this personalized growth path.

Adaptive learning platforms utilize learning analytics results to provide medical students with customized learning resources and exercises, ensuring that content matches their ability levels. Adaptive learning technology analyzes students’ learning data in-depth, such as learning behavior patterns, performance trends, and personal interests, revealing the learning needs and characteristics of medical students. These insights enable personalized algorithms to create tailored learning paths for each student, enhancing the relevance and attractiveness of learning and promoting active student engagement and in-depth understanding (Cook and Artino, 2016). This makes medical education more aligned with individual differences, achieving precise teaching. This personalized learning support not only helps students absorb knowledge more effectively but also improves the adaptability and efficiency of the learning process through instant feedback and dynamic adjustment of learning difficulty. Such a support mechanism contributes to better academic achievements for students in medical education.

The application effects of adaptive learning technology are assessed through continuous learning data analysis, which not only verifies its effectiveness in medical education but also reveals its positive impact on students’ learning outcomes, motivation, and experience (Cook and Artino, 2016). Comparative studies with traditional teaching methods further highlight the advantages of adaptive learning, providing an empirical basis and innovative direction for the continuous improvement of medical education.

## 7 DADA-driven educational improvement

In this study, “DADA” (Data-Driven Adaptive Learning) encompasses a broad range of technology fields related to educational data, with a focus on data mining and application. This concept extends beyond current Educational Data Mining (EDM) practices, anticipating future advancements in educational technology data mining and application (Viberg et al., 2018; Cristobal and Sebastian, 2024). DADA emphasizes the critical role of data in educational innovation, providing a comprehensive framework to address potential future developments in this field.

As a core component of DADA, Educational Data Mining (EDM) aims to extract patterns and trends from educational contexts to inform practice. EDM employs data mining techniques and methods such as machine learning, statistical analysis, and natural language processing to derive actionable insights from educational data, uncovering hidden patterns in large datasets related to medical students’ learning (Zawacki-Richter et al., 2019; Chen et al., 2020). This process supports the improvement and optimization of medical education, providing reliable scientific evidence for educational decision-making.

EDM enhances teaching methods, personalizes learning experiences, and informs educational policy formulation through data visualization and big data processing technologies (Yovanoff et al., 2017). It makes complex data comprehensible and uses predictive analytics to forecast student outcomes. Additionally, EDM conducts detailed analyses of medical students’ learning activities, time investment, and resource utilization, revealing their learning habits and efficiency (Been, 2012). These analyses assist educators in designing teaching strategies that better meet student needs, enhancing the relevance and effectiveness of medical education. EDM also delves into students’ academic performance data, identifying key factors affecting grades, and helping educators establish more accurate assessment systems to provide more effective learning support (Ifenthaler et al., 2019).

Through educational data mining technology, we can identify deficiencies in teaching methods and bottlenecks in student learning. These insights offer valuable information for educators, enabling them to adjust teaching content, methods, and resource allocation to achieve personalized learning, thereby improving the overall quality of medical education and student learning outcomes (Shahiri et al., 2015; Lu et al., 2018).

## 8 Conclusion and outlook

**Figure 2:**
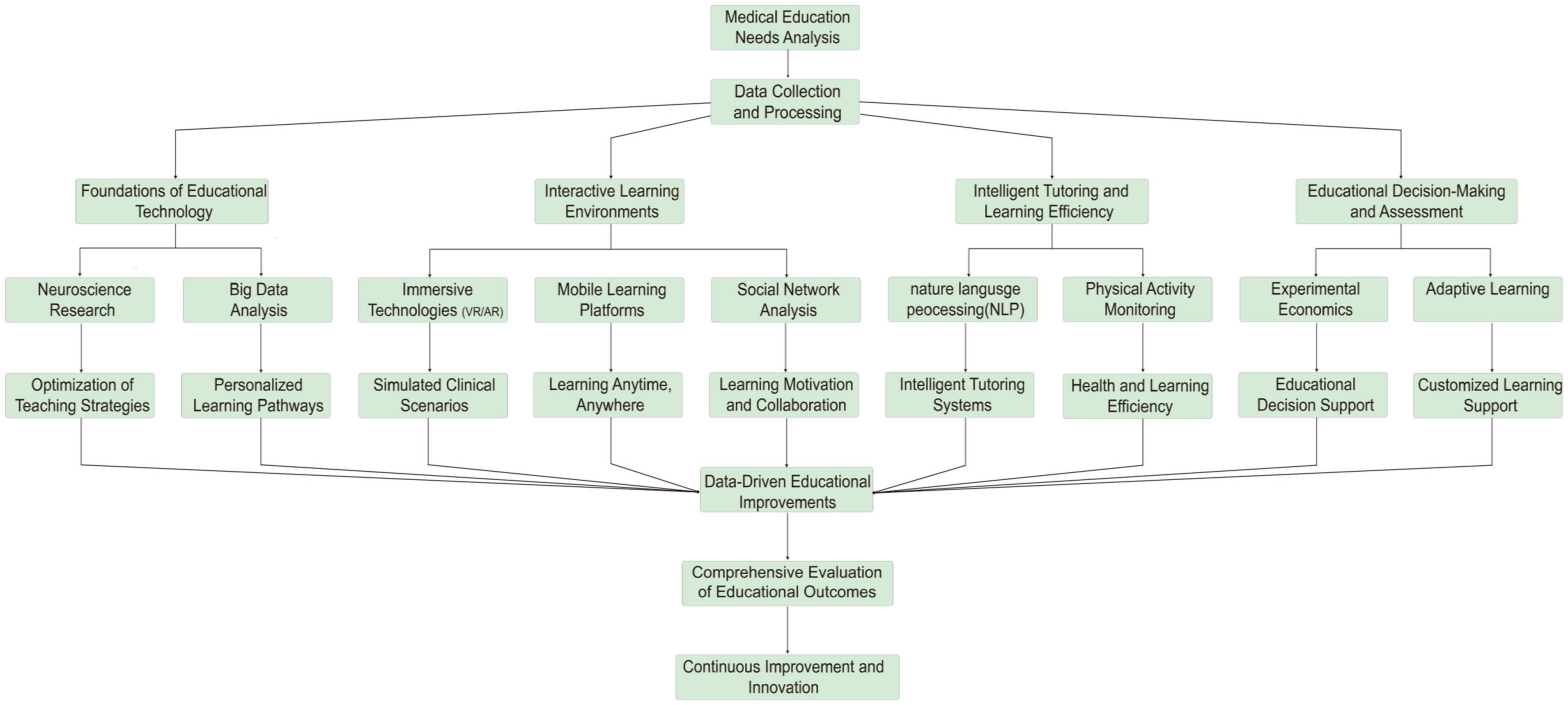
Integrative workflow of medical education technologies.

The integration of advanced technologies in medical education has been a transformative force, as underscored by the foundational contributions of neuroscience strategies (Carew and Magsamen, 2010) and personalized learning. These technologies have enabled interactive learning environments, such as immersive technologies and mobile learning, which have enhanced intelligent tutoring and learning efficiency. The data and tools provided by these advancements have facilitated educational decision-making and assessment, leading to a continuous optimization of educational practices and policy evaluation through data-driven improvements. This progression illustrates a logical evolution from basic research to practical application, and from decision-making to refinement, forming a cyclical optimization logic (Figure 2).

**Figure 3:**
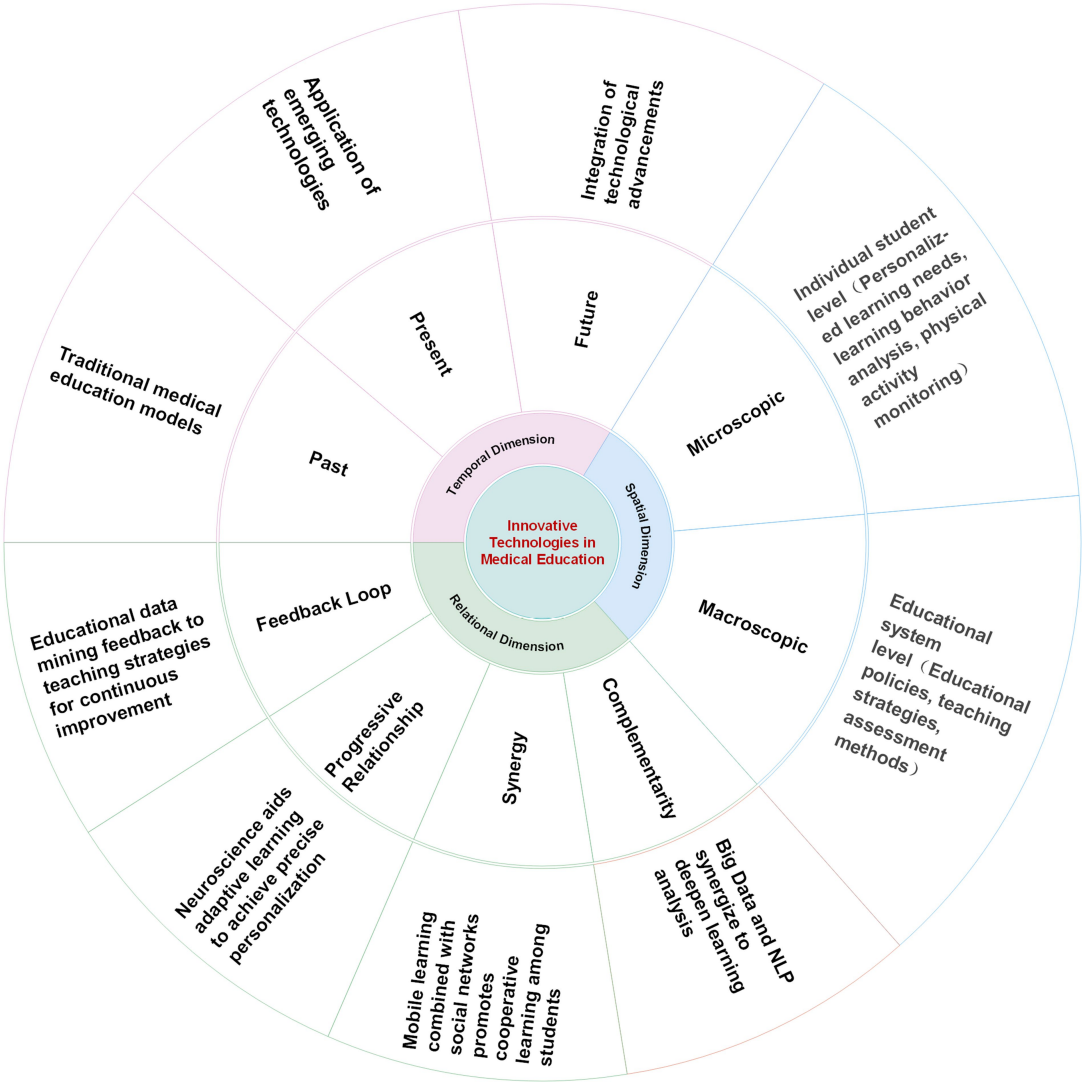
A multidimensional perspective on technological innovations in medical education.

Our multidimensional analysis has revealed the comprehensive impact of technological innovation on medical education, spanning from historical teaching paradigms to future trends, and from individual student needs to broader educational policies. The relational dimension of our analysis has highlighted the synergies among different technologies, which are crucial for the progressive optimization of educational strategies. This analysis not only deepens our understanding of technological transformations in medical education but also offers new perspectives and strategies for innovative practices in the field (Figure 3).

In assessing the impact of these technologies, we have identified both their advantages and challenges (Table 1). While each technology has the potential to modernize medical education, they also present challenges related to data quality, privacy protection, and technological adaptability. Therefore, it is imperative for educators and policymakers to address these challenges seriously to ensure the enhancement of educational quality.

**Table 1:** Advantages and challenges of key technologies.

Technology	Advantages	Challenges
VR (Virtual Reality)	Provides immersive clinical scenarios; enhances learning engagement	High equipment cost; technical complexity; potential issues with data privacy and security
Data Analytics	Enables personalized learning pathways; optimizes teaching strategies	Requires high-quality data; raises concerns about data privacy and security; demands advanced analytical skills
Mobile Learning	Facilitates anytime, anywhere learning; promotes learner autonomy	Requires reliable internet connection; may face accessibility and connectivity issues
NLP (Natural Language Processing)	Enhances interactive learning experiences; supports intelligent tutoring systems	Limited by complexity of natural language; requires advanced technical skills for effective implementation
Social Network Analysis	Strengthens learning motivation and collaboration; analyzes social dynamics	Requires understanding of social network structures; demands technical skills for network analysis
Adaptive Learning Systems	Personalizes learning experiences; adjusts to individual learner needs	Requires sophisticated algorithms; may face challenges in adapting to diverse learning styles
Experimental Economics	Scientific assessment of educational decisions, simulation of real decision-making challenges, research on incentive mechanisms	Discrepancy between experimental design and reality, participant selection bias, experimental costs
Physical Activity Monitoring	Enhances health and learning efficiency; monitors physical activity levels	Requires continuous monitoring; raises privacy and security concerns; demands user compliance

**Figure 4:**
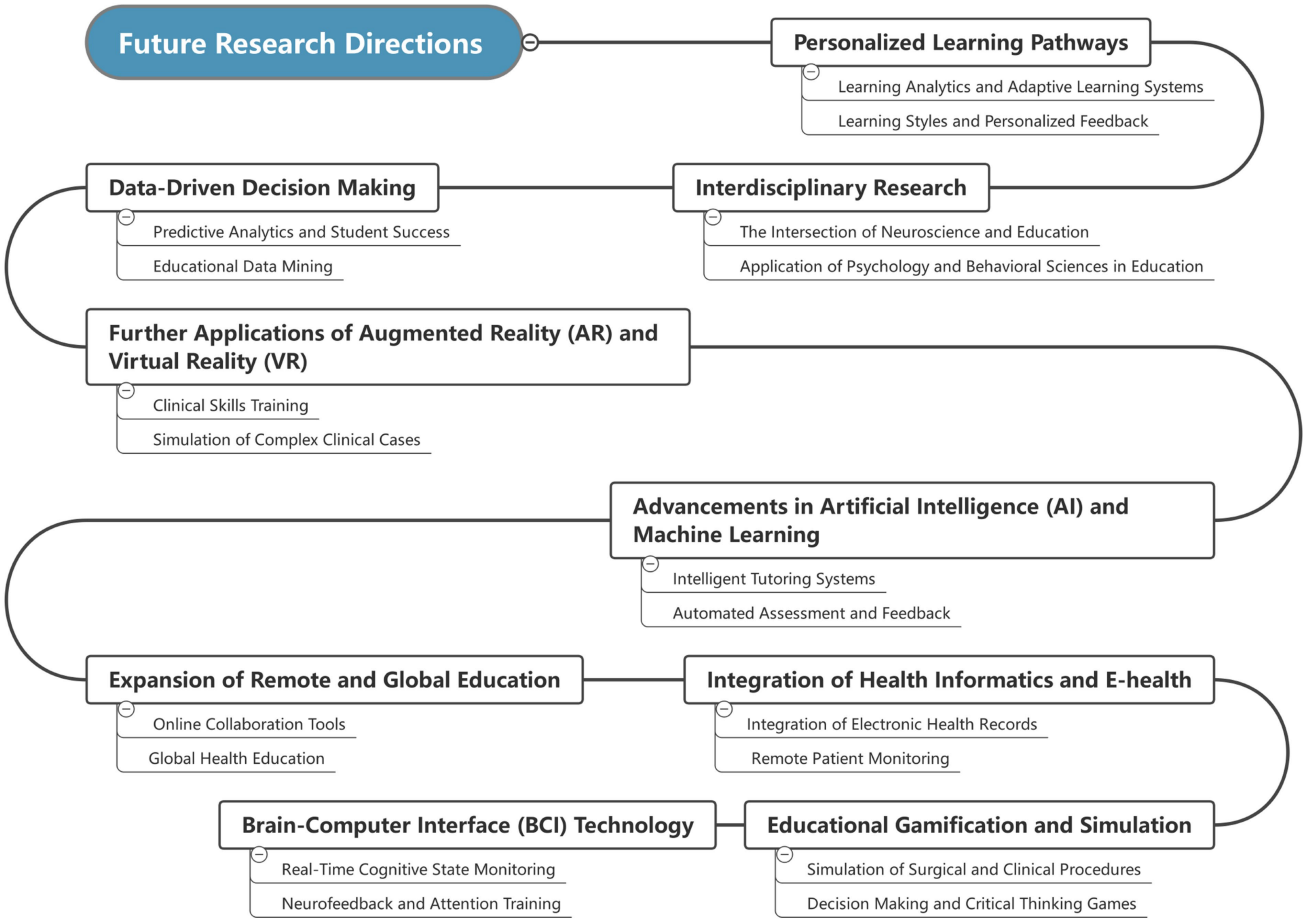
Future research directions in medical education technology.

Looking ahead, the development of educational technology is recognized as a continuously evolving process that necessitates ongoing research and innovation. Future research directions in educational technology should focus on further integration and optimization of technology (Juli et al., 2023; Robert, 2024), as well as the exploration of new methods and strategies in educational practice. These research directions will guide us toward a deeper understanding of how technology shapes the educational environment and achieves more effective learning and teaching (Figure 4).

With the convergence of artificial intelligence and machine learning, intelligent teaching systems will be able to monitor students’ learning progress in real-time and provide feedback. Policymakers must balance the advantages of technology with potential risks, ensuring that technological applications genuinely enhance educational quality. Continuous research and practice will aid in discovering more effective evaluation methods, optimizing educational strategies, and cultivating professional talents to meet future medical needs. Notably, the development of brain-computer interface (BCI) technology will offer a new perspective in medical education, providing real-time feedback on students’ cognitive states to educators, optimizing teaching strategies, and improving learning efficiency (Wan et al., 2024). As technology advances, medical education is poised to become more personalized, efficient, and innovative.

To conclude, our review has highlighted the significant potential of technological integration in medical education and has identified key areas for future research. We advocate for a collaborative approach that brings together educators, policymakers, and technologists to harness the power of technology for the betterment of medical education and the preparation of future healthcare professionals.

## References

[ref6] Ahmadian S. Rostami M. Farrahi V. Oussalah M. (2024). A novel physical activity recognition approach using deep ensemble optimized transformers and reinforcement learning. Neural Netw. 173:106159. doi: 10.1016/j.neunet.2024.106159, PMID: 38342080

[ref7] Al-bedaery R. Baig S. Khare Y. Sullivan-Mchale J. (2024). Humanising case-based learning. Med. Teach. 46, 1348–1355. doi: 10.1080/0142159X.2024.2308066, PMID: 38285885

[ref9] Baba K. Faddouli N.-E. E. Cheimanoff N. (2024). Mobile-optimized AI-driven personalized learning: a case study at Mohammed VI polytechnic university. Int. J. Interactive Mobile Technol. (iJIM) 18, 81–96. doi: 10.3991/ijim.v18i04.46547

[ref10] Bandura A. (Ed.). (1986). Social foundations of thought and action: A social cognitive theory. Englewood Cliffs, NJ: Prentice-Hall.

[ref11] Been W. (2012). Learning analytics: the new black. Available online at: http://www.mendeley.com/catalog/learning-analytics-new-black/ (Accessed November 27, 2024).

[ref45] Bi W. Cheng X. Xu B. Sun X. Xu L. Shen H. (2023). Bridged-GNN: Knowledge Bridge Learning for Effective Knowledge Transfer. In: Proceedings of the 32nd ACM International Conference on Information and Knowledge Management. 1–11. doi: 10.1145/3583780.3614796

[ref13] Carew T. J. Magsamen S. H. (2010). Neuroscience and education: an ideal Partnership for Producing Evidence-Based Solutions to guide 21(st) century learning. Neuron 67, 685–688. doi: 10.1016/j.neuron.2010.08.028 20826300

[ref14] Cecconi C. Adams R. Cardone A. Declaye J. Silva M. Vanlerberghe T. (2025). Generational differences in healthcare: the role of technology in the path forward. Front. Public Health 13:1546317. doi: 10.3389/fpubh.2025.1546317, PMID: 40078753 PMC11897013

[ref15] Cevallos N. Zukotynski B. Greig D. Silva M. Thompson R. M. (2022). The utility of virtual reality in orthopedic surgical training. J. Surg. Educ. 79, 1516–1525. doi: 10.1016/j.jsurg.2022.06.007, PMID: 35821110 PMC10364838

[ref16] Chen L. Chen P. Lin Z. (2020). Artificial intelligence in education: a review. IEEE Access 8, 75264–75278. doi: 10.1109/ACCESS.2020.2988510

[ref17] Chkhikvadze T. Kay C. Ramai D. (2024). Gastroenterological education: do digital data depositories make a difference? Dig. Dis. Sci. 69, 667–669. doi: 10.1007/s10620-024-08275-2, PMID: 38334935

[ref18] Cook D. A. Artino A. R. (2016). Motivation to learn: an overview of contemporary theories. Med. Educ. 50, 997–1014. doi: 10.1111/medu.13074, PMID: 27628718 PMC5113774

[ref29] Cristobal R. Sebastian V. (2024). Educational data mining and learning analytics: an updated survey - romero - 2020 - WIREs data min. Knowl. Discov. - Wiley online library. 10, 1–21. doi: 10.1002/widm.1355

[ref19] De Hei M. S. A. Strijbos J. W. Sjoer E. Admiraal W. (2015). Collaborative learning in higher education: lecturers’ practices and beliefs. Res. Pap. Educ. 30, 232–247. doi: 10.1080/02671522.2014.908407

[ref21] Fromm C. A. Huxlin K. R. Maddox R. K. Polonenko M. J. Diaz G. J. (2023). Multisensory perceptual learning in virtual reality may facilitate transfer to untrained locations but is impacted by training procedures.

[ref22] George T. M. Rastogi M. de Cothi W. Clopath C. Stachenfeld K. Barry C. (2024). RatInABox, a toolkit for modelling locomotion and neuronal activity in continuous environments. eLife 13:e85274. doi: 10.7554/eLife.85274, PMID: 38334473 PMC10857787

[ref23] Gurses M. E. Gonzalez-Romo N. I. Xu Y. Mignucci-Jiménez G. Hanalioglu S. Chang J. E. (2024). Interactive microsurgical anatomy education using photogrammetry 3D models and an augmented reality cube. J. Neurosurg. 141, 17–26. doi: 10.3171/2023.10.JNS23516, PMID: 38277660

[ref1] Harden R. M. Laidlaw J. W. (2012). Essential Skills for a Medical Teacher - 1st Edition | Elsevier Shop Available online at: https://shop.elsevier.com/books/essential-skills-for-a-medical-teacher/harden/978-0-7020-4582-0 (Accessed October 28, 2024).

[ref25] Huang D.-L. Zeng Q. Xiong Y. Liu S. Pang C. Xia M.. (2024). A combined manual annotation and deep-learning natural language processing study on accurate entity extraction in hereditary disease related biomedical literature. Interdiscip. Sci.: Comput. Life Sci. 17, 617–629. doi: 10.1007/s12539-024-00605-2 PMC11289304 38340264

[ref24] Ifenthaler D. Mah D. K. Yau Y. K. (Eds.). (2019). Utilising learning analytics for study success: reflections on current empirical findings. Cham: Springer.

[ref8] Juli K. Ela K. Deepak K. (2023). A structured analysis to study the role of machine learning and deep learning in the healthcare sector with big data analytics. Arch. Comput. Methods Eng. 31, 2001–2027. doi: 10.1007/s11831-023-09915-y PMC10064607 37359744

[ref27] Kade S. A. Toit S. A. du Danielson C. T. Schweizer S. Morrison A. S. Ong D. C. (2024). Aberrant cognitive empathy in individuals with elevated social anxiety and regulation with emotional working memory training. Cognit. Emot. 38, 259–275. doi: 10.1080/02699931.2024.2314981 38349272

[ref28] King Z. D. Yu H. Vaessen T. Myin-Germeys I. Sano A. (2024). Investigating receptivity and affect using machine learning: ecological momentary assessment and wearable sensing study. JMIR Mhealth Uhealth 12:e46347. doi: 10.2196/46347, PMID: 38324358 PMC10882474

[ref30] Kolstad M. Yamaguchi N. Babic A. Nishihara Y. (2020). “Integrating socially assistive robots into Japanese nursing care” in The importance of health informatics in public health during a pandemic. Eds. Mantas J. Hasman A. Househ M. S. Gallos E. (Amsterdam: IOS Press), 183–186. 10.3233/SHTI200524 32604631

[ref31] Kusurkar R. A. Croiset G. Galindo-Garré F. Ten Cate O. (2013). Motivational profiles of medical students: association with study effort, academic performance and exhaustion. BMC Med. Educ. 13:87. doi: 10.1186/1472-6920-13-87, PMID: 23782767 PMC3691760

[ref32] Lamb A. McKinney B. Frousiakis P. Diaz G. Sweet S. (2023). A comparative study of traditional technique guide versus virtual reality in Orthopedic trauma training. Adv. Med. Educ. Pract. 14, 947–955. doi: 10.2147/AMEP.S395087, PMID: 37693298 PMC10487700

[ref33] Lewis K. O. Popov V. Fatima S. S. (2024). From static web to metaverse: reinventing medical education in the post-pandemic era. Ann. Med. 56:2305694. doi: 10.1080/07853890.2024.2305694, PMID: 38261592 PMC10810636

[ref34] Li S. Hong Y.-C. Craig S. D. (2023). A systematic literature review of social learning theory in online learning environments. Educ. Psychol. Rev. 35:108. doi: 10.1007/s10648-023-09827-0

[ref35] Liu J. Zhao Z. Wu N. Wang X. (2024). Research on the structure function recognition of PLOS. Front. Artif. Intell. 7:101419. doi: 10.3389/frai.2024.1254671, PMID: 38327668 PMC10847351

[ref36] Lohre R. Leveille L. Goel D. P. (2021). Novel application of immersive virtual reality simulation training: a case report. JAAOS Glob. Res. Rev. 5:114. doi: 10.5435/JAAOSGlobal-D-21-00114, PMID: 34807871 PMC8604004

[ref37] Lu O. H. T. Huang A. Y. Q. Huang J. C. H. Lin A. J. Q. Ogata H. Yang S. J. H. (2018). Applying learning analytics for the early prediction of students’ academic performance in blended learning. Educ. Technol. Soc. 21, 220–232. Available at: https://www.jstor.org/stable/10.2307/26388400

[ref38] Margalit A. Suresh K. V. Marrache M. Lentz J. M. Lee R. Tis J.. (2022). Evaluation of a slipped capital femoral epiphysis virtual reality surgical simulation for the orthopaedic trainee. J. Am. Acad. Orthop. Surg., Glob. Res. Rev. 6:e22.28. doi: 10.5435/JAAOSGlobal-D-22-00028 PMC9042586 35467580

[ref39] McGaghie W. C. Barsuk J. H. Wayne D. B. (2020). Comprehensive healthcare simulation: Mastery learning in health professions education. Cham: Springer Nature.

[ref20] Mibiti I. Serra D. (2022). Health workers’ behavior, patient reporting and reputational concerns: lab-in-the-field experimental evidence from Kenya. Exp. Econ. 25, 514–556. doi: 10.1007/s10683-021-09721-y

[ref3] Mohammed A. MD Fahad M. A. MD. (2025). Generative artificial intelligence and the personalization of health professional education: A narrative review - PubMed. Available online at: https://pubmed.ncbi.nlm.nih.gov/39093806/ (Accessed October 29, 2024). 10.1097/MD.0000000000038955 PMC11296413 39093806

[ref40] Moulaei K. Sharifi H. Bahaadinbeigy K. Dinari F. (2024). Efficacy of virtual reality-based training programs and games on the improvement of cognitive disorders in patients: a systematic review and meta-analysis. BMC Psychiatry 24:116. doi: 10.1186/s12888-024-05563-z, PMID: 38342912 PMC10860230

[ref41] Mugahed A. R. W. Shahizan O. M. Lizawati M. Y. (2015). Exploring the factors that affect student satisfaction through using E-learning in Malaysian higher education institutions. Mediterr. J. Soc. Sci. 6, 299–310. doi: 10.5901/mjss.2015.v6n4s1p299

[ref12] Noah S. O. Katherine L. M.-W. Anne M. C. Brooke N. C. Daniel L. D. Thomas D. P.. (2024). A virtual reality paradigm with dynamic scene stimuli for use in memory research. Behav. Res. Methods. 56, 1–17. doi: 10.3758/s13428-023-02243-w PMC11018716 37845424

[ref44] Penaud S. Yeh D. Gaston-Bellegarde A. Piolino P. (2023). The role of bodily self-consciousness in episodic memory of naturalistic events: an immersive virtual reality study. Sci. Rep. 13:17013. doi: 10.1038/s41598-023-43823-2, PMID: 37813899 PMC10562507

[ref2] Rachel H. E. Janet C. David T. Maureen T. (2015). Exploring digital professionalism: Medical teacher: Vol 37, No 9 - Get Access. Available online at: https://www.tandfonline.com/doi/full/10.3109/0142159X.2015.1044956 (Accessed October 29, 2024).

[ref46] Richard B. Friedman B. (1984). The impact of technology on medical education. J. Educ. Technol. Syst. 13, 137–141.

[ref43] Robert C. Optimising, generalising and integrating educational practice using neuroscience | Npj science of learning (2024). Available online at: https://www.nature.com/articles/npjscilearn201612 (Accessed November 28, 2024). 10.1038/npjscilearn.2016.12 PMC6380379 30792897

[ref47] Ruichen J. (2024). The impact of mobile social networking motivation on resilience of college students——The mediating effect of personality. Huzhou, Zhejiang, China: J. Huzhou Univ.

[ref48] Ryan R. M. Deci E. L. (2000). Self-determination theory and the facilitation of intrinsic motivation, social development, and well-being. Am. Psychol. 55, 68–78. doi: 10.1037/0003-066X.55.1.68, PMID: 11392867

[ref49] Ryan R. M. Deci E. L. (2020). Ntrinsic and extrinsic motivations in psychology and education: A self-determination theory perspective. New York: Routledge, 1–18.

[ref50] Shahiri A. M. Husain W. Rashid N. A. (2015). A review on predicting student’s performance using data mining techniques. Procedia Comput. Sci. 72, 414–422. doi: 10.1016/j.procs.2015.12.157

[ref51] Stubbing E. A. Helmich E. Cleland J. (2019). Medical student views of and responses to expectations of professionalism. Med. Educ. 53, 1025–1036. doi: 10.1111/medu.13933, PMID: 31509286

[ref52] Suau Q. Bianchini E. Bellier A. Chardon M. Milane T. Hansen C. (2024). Current knowledge about ActiGraph GT9X link activity monitor accuracy and validity in measuring steps and energy expenditure: a systematic review. Sensors 24:825. doi: 10.3390/s24030825, PMID: 38339541 PMC10857518

[ref53] Sweller J. (2020). Cognitive load theory and educational technology. Educ. Technol. Res. Dev. 68, 1–16. doi: 10.1007/s11423-019-09701-3

[ref54] Sweller J. van Merriënboer J. J. G. Paas F. (2019). Cognitive architecture and instructional design: 20 years later. Educ. Psychol. Rev. 31, 261–292. doi: 10.1007/s10648-019-09465-5

[ref55] Teo Z. L. Jin L. Liu N. Li S. Miao D. Zhang X.. (2024). Federated machine learning in healthcare: a systematic review on clinical applications and technical architecture. Cell Rep. Med. 5:101589. doi: 10.1016/j.xcrm.2024.101419, PMID: 38340728 PMC10897620

[ref56] Twenge J. M. (2017). Why today’s super-connected kids are growing up less rebellious, more tolerant, less happy—And completely unprepared for adulthood. 1st Edn. New York: Atria Books.

[ref57] Vanslambrouck S. Zhu C. Lombaerts K. Philipsen B. Tondeur J. (2018). Students’ motivation and subjective task value of participating in online and blended learning environments. Internet High. Educ. 36, 33–40. doi: 10.1016/j.iheduc.2017.09.002

[ref58] Viberg O. Hatakka M. Bälter O. Mavroudi A. (2018). The current landscape of learning analytics in higher education. Comput. Hum. Behav. 89, 98–110. doi: 10.1016/j.chb.2018.07.027

[ref59] Wan C. Pei M. Shi K. Cui H. Long H. Qiao L. (2024). Toward a brain–neuromorphics interface. Adv. Mater. 36:e2311288. doi: 10.1002/adma.202311288, PMID: 38339866

[ref61] Wang R. Zheng N. Liang Y. Cui H. Ren T. Xing W.. (2025). Multi-perspective analysis of daVinci surgical virtual reality training: a prospective randomized controlled study. J. Robot. Surg. 19:221. doi: 10.1007/s11701-025-02309-1, PMID: 40377751 PMC12084236

[ref62] Wartman S. A. Combs C. D. (2019). Reimagining medical education in the age of AI. AMA J. Ethics 21, E146–E152. doi: 10.1001/amajethics.2019.146 30794124

[ref42] Yang L. Xiao-Kang M. Wen-Zhen S. Ya-Qun L. Chao T. Si-Si D.. (2024). Mir-34a/TAN1/CREB axis engages in alleviating oligodendrocyte trophic factor-induced myelin repair function and astrocyte-dependent neuroinflammation in the early stages of Alzheimer’s disease: the anti-neurodegenerative effect of treadmill exercise. Neurochem. Res. 49, 1105–1120. doi: 10.1007/s11064-024-04108-w 38289520

[ref63] Yovanoff M. Pepley D. Mirkin K. Moore J. Han D. Miller S. (2017). Personalized learning in medical education: designing a user interface for a dynamic haptic robotic trainer for central venous catheterization. Proc. Hum. Factors Ergon. Soc. Annu. Meet. 61, 615–619. doi: 10.1177/1541931213601639, PMID: 29123361 PMC5675531

[ref26] Zachary F. Emerson H. Katalin T. Richard N. (2023). Silences, spikes and bursts: three-part knot of the neural code – Friedenberger. J. Physiol - Wiley Online Library. 601, 5165–5193. doi: 10.1113/JP281510 37889516

[ref64] Zawacki-Richter O. Marín V. I. Bond M. Gouverneur F. (2019). Systematic review of research on artificial intelligence applications in higher education – where are the educators? Int. J. Educ. Technol. High. Educ. 16:39. doi: 10.1186/s41239-019-0171-0

[ref65] Zhen N. He H. Jinyu W. (2013). Adaptive learning in tracking control based on the dual critic network design. IEEE Trans. Neural Netw. Learn. Syst. 24, 913–928. doi: 10.1109/tnnls.2013.2247627 24808473

